# Characterization of *Staphylococcus aureus* isolated from patients with burns in a regional burn center, Southeastern China

**DOI:** 10.1186/s12879-018-2955-6

**Published:** 2018-01-25

**Authors:** Kaisen Chen, Shirong Lin, Peiqun Li, Qiuyue Song, Dong Luo, Tao Liu, Lingbing Zeng, Wei Zhang

**Affiliations:** 1 Department of clinical laboratory, The first Affiliated Hospital of Nanchang university, Nanchang, 330006 China; 2 Department of emergency, The first Affiliated Hospital of Nanchang University, Nanchang, 330006 China; 3 The College of Public Health of Nanchang University, Nanchang, 330006 China; 4 Department of respiration, The first Affiliated Hospital of Nanchang University, Nanchang, 330006 China

**Keywords:** *S. aureus*, Molecular epidemiology, Burns, China

## Abstract

**Background:**

*S.aureus* is a predominant pathogen that causes infection in critically ill patients, but little information exists regarding the characterization of *S. aureus* from different sources in burn patients in southeastern China.

**Methods:**

We enrolled 125 patients with *S. aureus* infection in burns center between Jan 2014 and Dec 2015. *S. aureus* isolates were characterized by antimicrobial susceptibility test, toxin gene detection, and molecular typing with multilocus sequence type, staphylococcal protein A (spa) type, and staphylococcal cassette chromosome mec (SCCmec) type.

**Results:**

Sixty-eight MRSA were isolated from SSTI and 31 from non-SSTI patients, respectively. Overall, the drug-resistant ability of *S. aureus* isolated from SSTI was higher than that from non-SSTI groups. SCCmecIII-CC239-t030 was the most common clone (38 from SSTIs, and 8 from non-SSTIs). *Seg* was the most common enterotoxin gene (21 from SSTIs and 33 from non-SSTIs). Isolates from SSTIs was more likely to carry seb (*P* = 0.04), while those from non-SSTIs tended to carry *sea* and *seg* (*P* = 0.002 and 0.01, respectively). Although isolates carried four hemolysin genes, there was no significant difference between them (*P* > 0.05).

**Conclusion:**

SCCmecIII-CC239-t030 was the most common clone in Jiangxi burns center, China. The molecular characterization of *S. aureus* was quite different between SSTI and non-SSTI groups.

## Background

Millions of people have burns every year in the world. Only in China, approximately 5000–10,000 peoples sustain burn injuries per 1 million, and 10% of them require medical intervention. Jiangxi province locates in southeastern region in China, and having numerous fireworks factories. Consequently, there are many thermal burns patients in local burn units, and millions of dollars having been consumed every year.

Skin is the first barrier of defense against microbial invasion, and it becomes more prone to infection once it gets burns. Many pathogens are responsible for burn wound infections, including *Staphylococcus* spp., *Enterococcus* spp., *Pseudomonas* spp., *Acinetobacter* spp., and fungi [1]. However, *Staphylococcus aureus* (*S.aureus*) remains a leading cause of infections in burn centers [2]. The emergence and transmission of methicillin-resistant *S. aureus* (MRSA) in burn centers results in some poor outcomes such as prolonged hospitalization, bacteremia or sepsis, even death, which require further prevention and treatment efforts [3].

The main task for successful clinical treatment of microorganism infection depends on the knowledge of the characteristics of these infecting strains and their antibiotic susceptibility profiles. As a result, identification of the antibiotic susceptibility patterns of different strains in the burns center provides more appropriate treatments and decreased expenses. In addition to antibiotic resistance, another important factor that leads to treatment failure in burns patients is the production of different virulence factors. For example, these Panton-Valentine Leukocidin (PVL*)*-producing strains of *S. aureus* are associated with increased lethality compared to the PVL-negative strains [4]. Skin and soft tissue infections (SSTIs) still have been taken as the primary source in burns patients [5, 6] and antibacterial ointment is usually taken as the mainstay of treatment, different from non-SSTIs patients who treated with injectable antibiotics in China. It is necessary to know different characteristics between SSTI and non-SSTI groups for empirical therapy at the early infectious stage.

Molecular typing of *S. aureus* is of significant value as a tool to control infection [7]. At present, there are several molecular typing methods, including multilocus sequence typing (MLST), staphylococcal protein A (spa) typing, and staphylococcal cassette chromosome mec (SCCmec) typing. In general, a combination of typing methods could provide more detailed genetic linkage among these strains.

A high rate of isolated *S. aureus* corresponds to a high infectious level in burn centers [1–3]. However, there are few studies on the characterization of high isolated *S. aureus* from patients with burns in Jiangxi province, China. Many factors have action on effective treatment and control of *S. aureus* or MRSA infections. As a result, it is extremely important to know the different pattern of antimicrobial resistance and the prevalent clone of *S. aureus* or MRSA for local optimal clinical control.

## Methods

### Bacterial isolates

This study included all clinical isolates submitted for culture and sensitivity test at the burn center of the First Affiliated Hospital of Nanchang University in China, from January 1, 2014s to December 31, 2015s. *S. aureus* isolates were identified by using the VITEK-2 compact (BioMérieux, France) GP colorimetric identification card. Resistance to oxacillin or cefoxitin was confirmed by VITEK-2 compact AST-GP67 (BioMérieux, France), with resistance defined as an oxacillin minimum inhibitory concentration (MIC) ≥ 4 μg/mL and cefoxitin MIC ≥8 μg/mL [8]. In the case of multiple *S. aureus* isolates from the same patient, the first detected resistant specimen was included. In total, 125 *S. aureus* isolates were characterized, including 75 from SSTIs and 50 from non-SSTIs. The isolated strains stored at − 80 °C were thawed and sub-cultured on BHI medium prior to DNA extraction.

### Antimicrobial susceptibility test (AST)

In addition to oxacillin and cefoxitin, other antimicrobials listed for routine reporting of *S. aureus* were included for AST by using VITEK-2 compact AST-GP67 (BioMérieux, France) test card for Gram-positive susceptibility. Antibiotics used for susceptibility test included benzylpenicillin, gentamicin, ciprofloxacin, levofloxacin, moxifloxacin, erythromycin, clindamycin, quinupristin/dalfopristin, linezolid, vancomycin, tetracycline, tigecycline, nitrofurantoin, rifampicin, and trimethoprim/sulfamethoxazole. The MIC interpretive standard or breakpoint values were set following the guidelines of Clinical and Laboratory Standard Institute M100-S23. *S. aureus* ATCC25923 was used as a quality control for MIC detection.

### Detection of toxin genes

Genomic DNA was extracted using a DNA extraction kit (Sangon Biotech, Shanghai) with lysostaphin, according to the manufacturer’s instructions. Several clinically significant toxin genes were detected by PCR [9, 10], including *lukS/F-PV* (encoding Panton-Valentine leukocidin), *tst* (encoding toxic shock syndrome toxin 1), *sea*-*see* and *seg*-*sej* (encoding staphylococcal enterotoxins SEA-SEE and SEG-SEJ), and *HL*a-*HL*g (encoding staphylococcal hemolysin).

### Detection of molecular types

All *S. aureus* isolates were investigated by multilocus sequence typing (MLST), and the products of seven housekeeping gene fragments were sequenced (Sangon Biotech, Shanghai) and compared with allele profiles from *S. aureus* database (www.mlst.net/). Spa and SCCmec typing of MRSA were performed as previously described [2] .

### Statistical analysis

The chi-square or Fisher’s exact test was used as appropriate using the software package SAS 8.2 (SAS Institute Inc., Cary, NC, USA). A two-sided *P* value of < 0.05 was considered statistically significant.

## Results

### Clinical data

The median age of burns patients was 26 years and 7 months (range: 3 months - 79 years), as the sex distribution of male/female was 69/56 (55.2%/44.8%). All enrolled patients were from the burns center. The infectious source for burns patients included SSTI (75/125, 60.0%), respiratory tract (21/125, 16.8%), blood (14/125, 11.2%), and others (15/125, 12.0%).

### Antimicrobial susceptibility test

Ninety-nine isolates (68 isolated from SSTIs and 31 from non-SSTIs) were confirmed as MRSA and 26 isolates (7 isolated from SSTIs and 19 from non-SSTIs) confirmed as methicillin-susceptible *S.aureus* (MSSA), respectively. All 125 isolates were susceptible to vancomycin, linezolid, daptomycin, and quinupristin/dalfopristin. 15 isolates (3 from SSTIs and 12 from non-SSTIs) were susceptible to penicillin. The antimicrobial susceptibilities of *S. aureus* isolated from SSTIs were almost equal to those from non-SSTIs. These findings were summarized in Table 1.

**Table 1:** Antimicrobial susceptibility profile of *S. aureus*

Antimicrobial	*S.aureus* in SSTI (*n* = 75)			*S.aureus* in non-SSTI (*n* = 50)		
	R(%)	I(%)	S(%)	R(%)	I(%)	S(%)
Penicillin G	93.3	0.0	6.7	84.0	0.0	16.0
Cefoxitin Screen	90.7	0.0	9.3	62.0	0.0	38.0
Oxacillin	88.0	0.0	12.0	60.0	0.0	40.0
Gentamicin	60.0	2.7	37.3	38.0	2.0	60.0
Ciprofloxin	62.7	2.7	34.6	64.0	2.0	34.0
Levofloxacin	42.7	1.3	56.0	52.0	4.0	44.0
Moxifloxacin	25.3	4.0	70.7	34.0	4.0	62.0
Clindamycin	78.7	2.7	18.6	54.0	2.0	44.0
Erythromycin	90.7	2.7	6.6	50.0	4.0	46.0
Tetracycline	72.0	1.3	26.7	50.0	2.0	48.0
Rifampicin	49.3	2.7	48.0	30.0	2.0	68.0
Quinupristin/Dalfopristin	0.0	0.0	100.0	0.0	0.0	100.0
Trimethoprim/Sulfamethoxazole	18.7	0.0	81.3	16.0	0.0	84.0
Tigecycline	1.3	0.0	98.7	2.0	0.0	98.0
Nitrofurantoin	0.0	0.0	100.0	0.0	0.0	100.0
Linezolid	0.0	0.0	100.0	0.0	0.0	100.0
Vancomycin	0.0	0.0	100.0	0.0	0.0	100.0

*SSTI*, skin and soft tissue infection

### Virulence factors

As shown in Table 2, *seg* (54/125, 43.2%) was the most frequently detected enterotoxin gene, of which 21 isolated from SSTIs and 33 isolates were from non-SSTIs. 19 isolates showed presence of *lukS/F-PV*, including 15 SSTIs and 4 non-SSTIs. No *seh*-positive isolates were detected. Staphylococcal *HLa* and *HLg* were the most frequently detected hemolysin genes, occurring in 118 isolates. *S. aureus* isolates from SSTIs were more likely to carry *sea* and *seg* (*P* = 0.002 and 0.01, respectively), whereas non-SSTI patients were more likely to carry *seb* (*P* = 0.04).

**Table 2:** Prevalence of virulence genes from 125 *S. aureus* isolates

Genes	Total (*n* = 125)	SSTI (*n* = 75)	non SSTI (*n* = 50)	*P* value
*lukS/F-PV*	19	15	4	0.112
sea	16	3	13	0.002
seb	19	16	3	0.042
sec	3	3	0	0.161
sed	5	2	3	0.372
see	1	1	0	0.415
seg	54	21	33	0.009
seh	1	0	1	0.223
sei	29	19	10	0.583
sej	6	3	3	0.626
tst	7	5	2	0.547
HLa	118	72	46	0.871
HLb	59	38	21	0.567
HLd	99	62	37	0.689
HLg	118	73	45	0.766

*SSTI*, skin and soft tissue infection; *lukS/F-PV*, gene encoding Panton-Valentine leukocidin; *sea-see* and *seg-sej*, gene encoding staphylococcal enterotoxins; *tst*, gene encoding toxic shock syndrome toxin 1; *HLa- HLg*, gene encoding staphylococcal hemolysin. *P*-value and two-sided *P*-value were calculated by the chi-square or Fisher’s exact test appropriately

### Molecular epidemiological characteristics

**Fig. 1:**
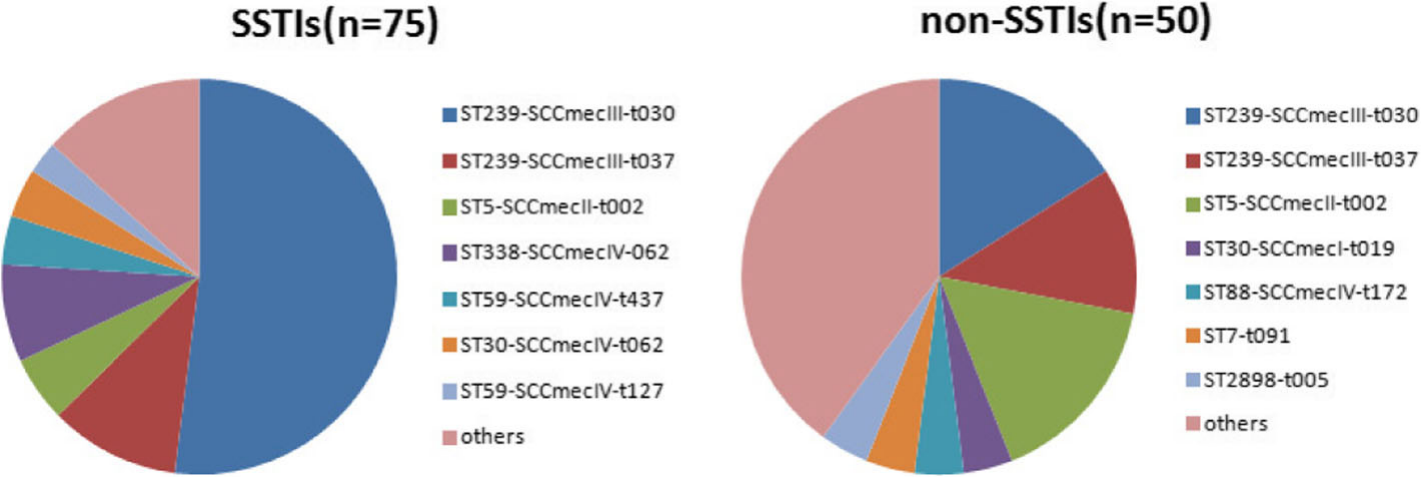
Distribution of major clones among 125 *S.aureus* isolates

Among 125 *S. aureus* isolates, 21 sequence types (STs) and 27 spa types were acquired (Table 3). We detected 4 SCCmec type I, 12 SCCmec type II, 61 SCCmec type III, 20 SCCmec IV, and 2 SCCmec type V. As shown in Fig. 1, MSSA isolates expressed greater diversity in molecular characteristics than MRSA; 43 clones were present in these isolates, including 10 shared clones.

**Table 3:** Distribution of major clones among 125 *S. aureus* isolates

Clone	CCs	Total	SSTIs	Non- SSTIs	Virulence genes
ST239-SCCmecIII-t030	8	47	38	8	LukS/F-PV(11),Sea(6), seb(4), sed(3), seg(27), sei(16), sej(3), tst(2), HLa(46), HLb(22), HLd(46), HLg(46)
ST239-SCCmecIII-t037	8	14	8	6	LukS/F-PV(2),sea(2), seb(2),sed(1), seg(5), sei(3) sej(1), HLa (13), HLb(8), HLd(11), HLg(13)
ST338-SCCmecIV-062	59	8	7	1	sea(1), seb(3), sec(1), sei(2), seg(1), tst(2), HLa(8), HLb(3), HLd(6), HLg(7)
ST5-SCCmecII-t002	5	12	4	8	sea(4), sed(1), seg(8), sei(1), HLα(10), HLβ(4), HLγ(10), HLδ(11)
ST59-SCCmecIV-t127	59	2	2	0	seb(1),see(1), HLa(1), HLb(1),HLg(2)
ST59- SCCmecIV-t437	59	3	3	0	seb(1),tst(1), HLa(3), HLb(2), HLd(2), HLg(3)
ST30-SCCmecI-t019	30	2	0	2	seb(1), seg(2), HLa(2), HLb(1),HLd(1), HLg(2)
ST30-SCCmecIV-t062	30	4	3	1	sea(1), seb(3), sec(1), sej(1), HLa(2), HLb(2), HLd(2), HLg(3)
ST88-SCCmecIV-t172	88	2	0	2	LukS/F-PV(1), Seb(1), seg(1), HLa(2), HLb(1), HLd(2), HLg(2)
ST398-SCCmecI-t034	398	2	1	1	Luks/F-PV(1), Seb(1), sej(1), HLa(2), HLb(1), HLd(2), HLg(2)
ST88-SCCmecV-t3740	88	1	0	1	Luks/F-PV(1), seg(1), HLa(1), HLb(1), HLg(1)
ST45-SCCmecV-t1081	45	1	0	1	seg(1), HLa(1), HLb(1), HLg(1)
ST88-IV-t002	88	1	1	0	Seb(1), sec(1), HLa(1), HLb(1), HLg(1) Luks/F-PV(1),sea(1), sed(1), sei(1), tst(1), HLa(2), HLb(1)
ST239-t30	8	2	1	1	HLd(2), HLg(2)
ST25-t037	25	2	1	1	Luks/F-PV(1), Seb(1), HLa(2), HLb(1), HLd(1), HLg(2)
ST7-t091	7	2	0	2	seg(2), sei(2), tst(1), HLa(2), HLb(1), HLd(2), HLg(2)
ST5-t002	5	1	1	0	sej(1), HLa(1), HLg(1)
ST6-t701	6	1	0	1	sea(1), seg(1),sei (2), HLa1), HLd(1), HLg(1)
ST188-t318	188	1	1	0	seg(2), sei(1), tst(1), HLa(1), HLb(1), HLd(1), HLg(1) LukS/F-PV(1), sea(1), seb(1), seh (1), HLa(2), HLb(1), HLd(1),
ST59-t163	59	2	1	1	HLg(2)
ST30-t045	30	1	0	1	tst(1), HLa(1), HLb(1), HLg(1)
ST2898-t005	30	2	0	2	sei(1), HLa(2), HLb(1), HLd(1), HLg(2)
ST50-t185	50	1	0	1	HLa(1), HLd(1), HLg(1)
ST25-t078	25	1	1	1	HLa(1), HLg(1)
ST1-t127	1	2	1	1	sea(1), HLa(2), HLb(1), HLd(1), HLg(1)
ST2155-t1451	121	1	0	1	tst(1), HLa(1), HLd(1), HLg(1)
ST5-t045	5	1	0	1	seg(1), HLa(1), HLb(1), HLd(1), HLg(1)
ST398-t571	398	1	0	1	seg(1), sej(1), HLa(1), HLb(1), HLd(1), HLg(1)
ST152-t002	152	1	0	1	sei(1), HLa(1), HLb(1), HLd(1), HLg(1)
ST17-t3741	17	1	0	1	HLa(1), HLb(1), HLg(1)
ST641	641	1	0	1	seg(1), HLa(1), HLd(1), HLg(1)
ST9-t878	9	1	1	0	seg(1),HLa(1), HLg(1)
ST45-t8232	45	1	0	1	HLa(1), HLd(1), HLg(1)

SSTI, skin and soft tissue infection

Twenty sequence types (STs) from 13 clonal complexes (CCs) were identified among all 125 *S. aureus* isolates. The most common ST was ST239 (63/125, 50.4%), followed by ST5 (14/125, 11.2%), ST59 (7/125, 5.6%), and ST30 (7/125, 5.6%). ST239 was detected in 44 isolates from SSTIs and 19 isolates from non-SSTIs, and ST59 was found in 6 isolates from SSTIs and 1 isolate from non-SSTI. The distribution of *S. aureus* in ST239 and ST5 was significantly different between SSTIs and non-SSTIs (*P* = 0.057 or 0.006); however, there was no difference in the distribution of ST59 and ST30 between isolates from SSTI and non-SSTI groups (*P* = 0.174, 0.111). ST239 was the most common ST among *lukS/F-PV*-positive isolates, including 11 isolates (10 MRSA and 1 MSSA) from SSTIs and 8 isolates (6 MRSA and 2 MSSA) from non-SSTIs. The other STs in *lukS/F-PV*-positive isolates were ST88 (2 MRSA), ST25 (1 MRSA), and ST398 (1 MRSA).

The different 26 spa type was identified in this study. t30 (49/125, 39.2%) was the most common spa type, followed by t037 (16/125, 12.8%) and t062 (14/125, 11.2%). t30 was detected in 39 isolates from SSTIs and in 10 isolates from non-SSTIs, and t037 was detected in 9 isolates from SSTIs and 7 isolates from non-SSTIs. The former displayed a significant difference between isolates from SSTIs or non-SSTIs (*P* = 0.014), while the latter did not (*P* = 0.774).

## Discussion

As a clinically important pathogen, *S. aureus* can cause many kinds of infections, such as SSTI, sepsis, pneumonia, and abscesses. As shown in the results section, there were 75 SSTIs, 14 blood or other bodily fluids, and 21 respiratory infection samples from *S. aureus* infected patients. Additionally, 68 isolates (15 community-acquired MRSA (CA-MRSA) and 53 hospital-acquired MRSA (HA-MRSA)) were obtained from SSTIs and 31 isolates (6 CA-MRSA and 25 HA-MRSA) from non-SSTIs. There was no significant difference between SSTI and non-SSTI groups in the occurrence of MRSA (*P* = 0.179). However, as shown in Table 1, *S.aureus* had higher resistance to erythromycin in SSTIs than that from non-SSTIs (*P* = 0.02), probably due to a high level of MRSA infection, which commonly integrates some important resistance genes [11]. Since its emergence in 1995 in Brazil, prevalence of ST239 has led to a high occurrence of hospital-acquired infection globally [12], as witnessed by *S. aureus* clone outbreaks in China, Africa, Australia, and America [13–15]. In this study, ST239 was found to be the most prevalent clone in the burn center (50.4%, 63/125), which is consistent to reports that ST239 is the most common clone in mainland China [16]. ST239 is usually associated with SSTIs disease [17], as a result, it was in expectation that patients with SSTIs had a higher proportion of infection caused by the ST239 clone than patients with non-SSTIs (*P* = 0.048). ST239 is mainly associated with staphylococcal cassette chromosome mec type III (ST239-SCCmecIII) [18]. As shown in Table 3, this study demonstrated evidence that more than half of the isolates from SSTIs were ST239-SCCmecIII-t030 or ST239-SCCmecIII-t037, and this number was higher than those isolates from non-SSTIs. ST239-SCCmecIII-t037 is closely related with ST239-SCCmecIII-t030, as the former is considered an ancestral ST239 spa type and has been gradually replaced by ST239-SCCmecIII-t030 in many places [19, 20]. ST5, found characterized in a total of 12 MRSA and 2 MSSA isolates, was the second most common ST in our study. ST5 has also been found to be among the most common genotype isolated from children with cystic fibrosis (CF) or nasal carriage [21, 22].

Comprehensively considered high antibiotic resistance and high virulence, some isolated *S. aureus* were a dangerous pathogen in this study. The harmfulness of *S. aureus* is partially determined by a series of virulence factors, which vary significantly according to different strains. As a result, to acquire valuable epidemiologic control in an endemic area, it is necessary to know the distribution of some important virulence factors. Staphylococcal enterotoxins (SEs) include a series of virulent factors associated with a severe disease, which usually causes food poisoning. SEs may suppress the motility of the human polymorphonuclear neutrophils through the inhibition of exoprotein expression, and allow *S. aureus* to invade and damage tissues. Although previous studies have confirmed that *sea* and *seb* are the most abundant toxin genes in clinical *S. aureus* isolates from patients in China [23], *seg* was the predominant SE gene detected in current study. There was significant difference observed between isolates from SSTIs and non-SSTIs (*P* = 0.009), which might represent regional characteristics. The study confirms that isolates from SSTIs predominantly carry *seb* (*P* = 0.042), which has been proved to be a hallmark of CA-MRSA [24]. There are four hemolysin genes in *S. aureus*, with the roles of which usually lead to the release of bacteriolytic enzyme that damages the surrounding cells by inhibiting blood cell function. HLa and HLb are the main toxins inducing pathological injury. Consistent with the report in Wenzhou, China [25], HLa, HLb, HLd, and HLg were all been detected in this study. These findings indicated that a high level of HLa, HLb, HLd, and HLg genes could be considered characteristic of MRSA strains in this region. There was no difference between isolates from SSTIs and non-SSTIs in these genes (*P* = 0.567–0.871, respectively). *LukS/F-PV* encodes Panton-Valentine leukocidin, an important virulent factor which usually damages neutrophils and is associated with SSTIs [26]. There is no connection between PVL-positive isolates among *S. aureus* isolated from SSTIs and those from non-SSTIs (*P* = 0.112), mostly because these strains originate from SSTIs.

## Conclusion

Our study indicated that the infectious rate of MRSA was high in burns center and SSTIs had higher resistant levels than non-SSTIs. Additionally, SSTIs have different virulent factors profiles compared with non-SSTIs. The most prevalent clone was ST239-SCCmecIII- t030 in the burn center.

## Abbreviations

CLSI: Clinical and Laboratory Standards Institute
MRSA: Methicillin-resistant *S.aureus*
MSSA: Methicillin-susceptibility *S.aureus*
SCCmec: Staphylococcal cassette chromosome mec
SSTI: Skin and soft tissue infection
